# Supplementary material for: Soil Reservoir Dynamics of Ophidiomyces ophidiicola, the Causative Agent of Snake Fungal Disease
Source: J Fungi (Basel). 2021 Jun 8;7(6):461. doi: 10.3390/jof7060461 (PMC8226778; doi:10.3390/jof7060461)
Supplement: Supplementary file 1 [file jof-07-00461-s001.zip › jof-1199253-supplementary.pdf]

Soil Reservoir Dynamics of *Ophidiomyces ophidiicola*, the Causative Agent of Snake Fungal Disease

LEWIS J. CAMPBELL[1,2,6], JOANNA BURGER[3], ROBERT T. ZAPPALORTI[4], JOHN F. BUNNELL[5], MEGAN E. WINZELER[1], DANIEL R. TAYLOR[1], JEFFREY M. LORCH[1,6]

1. U.S. Geological Survey – National Wildlife Health Center, WI 53711, USA
2. Department of Pathobiological Sciences, School of Veterinary Medicine, University of Wisconsin-Madison, WI 53706, USA
3. Division of Life Sciences, Rutgers University, NJ, USA
4. Herpetological Associates, Inc. NJ 08068, USA
5. New Jersey Pinelands Commission, NJ 08068, USA
6. CORRESPONDENCE: email, jlorch@usgs.gov; Lewiscampbell866@gmail.com

APPENDIX 1: SUPPLEMENTARY MATERIALS

Microbial Community Analysis - Methods

DNA extraction, amplification, and sequencing. - For each of the 10 experimental soils, 2 non-sterilized 300 mg aliquots were extracted using the Qiagen DNeasy PowerLyzer PowerSoil Kit (Hilden, Germany) following the manufacturer's standard protocol. Polymerase chain reaction was used to amplify 16S (v4 region; bacteria) and ITS2 (fungi) amplicons from each sample using the primer sets 515f and 806Rb (Gilbert et al. 2014) and ITS3KYO2 (Toju et al. 2012) and ITS4 (White et al. 1990),

respectively. All primers were appended with Illumina Nextera (Illumina, San Diego USA) compatible overhangs. Three replicate PCRs were performed for 16S and ITS2 per each extract. Each reaction consisted of 12.5 µl of Qiagen HotStar PCR master mix (Qiagen, Hilden, Germany), 0.5 µl each of 10 µM forward and reverse primers, 9.5 µl of sterile molecular grade water, and 2 µl of template DNA for a total reaction volume of 25 µl. Thermocycler conditions can be found in table S3. Post amplification, the three amplicon replicates for each DNA extract were pooled into one representative PCR product per soil extract. Pooled PCR products were cleaned using AMPure beads (Beckman Coulter, Brea, USA) following manufacture's standard protocol, barcoded with Illumina flowcell adapters following the protocol of Dunn et al. (2020), and sequenced in-house on an Illumina MiSeq system using V3 chemistry 600 cycle flow cells, generating paired end sequencing reads. One flow cell was used for each amplicon type. We included three negative controls on each sequencing run. Negative controls consisted of three extraction negatives (no soil), which were subsequently amplified for each amplicon type and prepared for sequencing simultaneously and following the same methods as our soil samples.

Sequence quality control and processing. - We used the R package DADA2 (Callahan et al. 2016a) to quality-screen and trim sequence reads. The following quality control steps were applied independently to our 16S and ITS2 datasets. We trimmed reads at the first appearance of a base with a quality score of two or lower. The first 10 bases of each read were removed and then forward reads were truncated at 240 bases in length and reverse reads at 200 bases in length. We also removed

reads with non-assigned bases (N) and reads mapping to the PhiX sequencing standard. We then applied DADA2 to detect sequence variants (SVs) and merged paired reads into single consensus reads, after which we removed chimeric sequences. The R package decontam (Davis et al. 2018) was then used to identify and remove contaminant sequence variants using sequence data generated from our negative controls. We assigned SVs to taxonomic groupings at the genus level or higher using the SILVA (v132.2) ribosomal RNA database (16S, Quast et al. 2013) and the UNITE (v8.1) fungal rRNA database (Kõljalg et al. 2013). We also removed reads that were not assigned a bacterial or fungal origin from the respective read sets at this stage. We further refined our dataset by removing all sequence variants that were unclassified at the phyla level, and ultra-rare phyla which occurred in only one sample, as these are likely erroneous sequencing artefacts (Callahan et al. 2016b). We conducted subsequent analyses using the R package phyloseq (McMurdie and Holmes 2013). As our intent was to interrogate broad patterns between soil microbial communities, rather than identify individual sequence variants of interest we amalgamated all SVs at the genus level. One 16S library for den 5 did not yield satisfactory sequencing data and was excluded from subsequent analysis.

Alpha diversity. - We also used phyloseq to compute alpha diversity metrics of the bacterial and fungal microbial communities of each soil sample. To ensure equal sampling effort we first rarefied our datasets down to the read levels of the least abundant sample per dataset. Our bacterial dataset was rarefied down to 28,629 reads per sample. Our fungal dataset was rarefied down to 38,595 reads per sample (Weiss

et al. 2017). We then computed the Shannon diversity indices of each soil's communities, and then converted this value into an effective number of species (ENS) value, following the recommendations of (Jost 2006). We also recorded the raw number of observed genera, to represent the species richness of each community. We investigated variation in alpha diversity based on soil location (hibernaculum vs topsoil), whether or not Oo was detected, and whether or not Oo grew during microcosm experiments. The distributions of both alpha diversity metrics were shown to be non-normal between all these groups using Shapiro-Wilk tests, therefore between group differences in alpha diversity were statistically assessed using generalised linear mixed effects models implemented in the R package lme4 (Bates et al. 2015). For both bacterial and fungal datasets models with ENS as the response variable were fitted using a Gaussian error distribution with a square root link function. Models with observed genera as the response variable were fitted with a Poisson error distribution with a square root link function. Soil type (topsoil versus hibernaculum), whether naturally occurring Oo was detected in the soil (yes versus no), and whether Oo grew in the soil during microcosm growth experiments (yes versus no) were fitted as fixed effects. To control for variation in diversity and richness attributable to variation between dens, den number was fitted as the sole random effect in all models. The tab_model function of the R package sjPlot (Lüdecke 2021) was used to compute the significance of each fixed effect from the global model. Model residuals were confirmed to be normally distributed and heteroskedastic.

Beta diversity. - We used the R package vegan (Oksanen et al. 2018) to quantify the between sample differences in microbial community composition (beta diversity). As vegan can accommodate non-integer data, rather than rarefy our datasets for this analysis, both datasets were instead converted into relative, proportional abundance values.

We produced two non-metric multi-dimensional scaling (NMDS) ordinations using Bray–Curtis dissimilarity of between-sample differences in bacterial and fungal communities. Ordinations were performed across two dimensions (k=2), ran for 1000 permutations, and yielded stress of fit values of 0.03 for bacterial and 0.06 for fungal communities. We tested our distance matrices for homogeneity of dispersion using the betadisper function of vegan and found that neither our bacterial nor our fungal datasets displayed heterogenous dispersion. We tested for differences in community composition between sample groups using a marginal permutational analysis of variance test (PERMANOVA) implemented using the adonis2 function in vegan. Our PERMANOVA modelled the differences in community structure between samples based on soil type, Oo detection, and Oo growth. Marginal PERMANOVA computes the significance of each variable of interest on community structure, accounting for variation explained by other variables; this negates issues related to variable ordering which impact other methods of PERMANOVA.

To detect differentially abundant genera between the sample groupings of interest as determined by PERMANOVA tests, we used the linear discriminant analysis effect size method (LEfSe; Segata et al. 2011). This analysis is a two-stage

process that first determines which genera are differentially enriched between comparison groups and then determines which of those differentially enriched genera are consistently represented among individuals within a group.

Microbial Community Analysis - Results

Sequencing data processing – bacterial communities.- In total, 4,761 SVs were identified in 16S rRNA reads from soils. When reads assigned to non-bacterial taxa were removed (n = 61) 4,700 bacterial taxa remained in the dataset. Twelve SVs were identified as potential contaminants and were excluded from the dataset giving a total of 4,688 bacterial taxa. Reads originating from each SV were compiled at the genus level, resulting in 301 representative unique genera accounting for 1,090,925 reads. One replicate sample of hibernaculum 4 failed to generate high quality sequencing reads and was excluded from our analyses.

Sequencing data processing – fungal communities. - A total of 2,581 taxa were identified in our ITS2 read dataset. No non-fungal organisms were detected. Two SVs were identified as potential contaminants and were excluded from the dataset giving a total of 2,579 fungal SVs. Reads originating from each SV were compiled at the genus level, resulting in 379 unique representative genera accounting for 1,323,115 reads.

Alpha diversity. - The results of our alpha diversity analysis are presented in full in the main manuscript.

Beta diversity.- The bacterial community of hibernaculum soils were dominated by sequence variants belonging to the genera *Massillia* and *Pseudarthrobacter*, whereas the native bacterial communities of topsoil samples were

dominated by sequence variants belonging to the genera *Cabelleronia, Conexibacter, Mucilaginibacter,* and *Mycobacterium* (Figure S2). The fungal communities of hibernaculum soils were dominated by sequence variants belonging to the genera *Pseudogymnoascus*, *Talaromyces*, *Heterobasidion* and *Helicodendron*. The fungal communities of topsoils were dominated by sequence variants belonging to the genera *Venturia*, *Cenococcum*, and *Inocybe* (Figure S3).

Disclaimer: Any use of trade, firm, or product names is for descriptive purposes only and does not imply endorsement by the U.S. Government.

Cited Literature

Bates, D., M. Maechler, B. Bolker, and S. Walker. 2015. Fitting Linear Mixed-Effects Models Using lme4. J. Stat. Softw. 67:1–48. DOI: https://doi.org/10.18637/jss.v067.i01.

Callahan, B.J., P.J. McMurdie, M.J. Rosen, A.W. Han, A.J.A. Johnson, and S.P. Holmes. 2016a. DADA2: High-resolution sample inference from Illumina amplicon data. Nat. Methods 13:581–583. DOI: https://doi.org/10.1038/nmeth.3869

Callahan, B.J., K. Sankaran, J.A. Fukuyama, P.J. McMurdie, and S.P. Holmes. 2016b. Bioconductor workflow for microbiome data analysis: From raw reads to community analyses [version 1; referees: 3 approved]. F1000Research 5:1–49. DOI: https://doi.org/10.12688/F1000RESEARCH.8986.1

Davis, N.M., D.M. Proctor, S.P. Holmes, D.A. Relman, and B.J. Callahan. 2018. Simple statistical identification and removal of contaminant sequences in marker-gene and metagenomics data. Microbiome 1–14.

Dunn, C.D., L.J. Campbell, E.M. Wallace, … T.L. Goldberg. 2020. Bacterial communities on the gills of bonefish (Albula vulpes) in the Florida Keys and The Bahamas show spatial structure and differential abundance of disease-associated bacteria. Mar. Biol. 167:1–11. DOI: https://doi.org/10.1007/s00227-020-03698-7

Gilbert, J.A., J.K. Jansson, and R. Knight. 2014. The Earth Microbiome project: Successes and aspirations. BMC Biol. 12:1–4. DOI: https://doi.org/10.1186/s12915-014-0069-1

Jost, L. 2006. Entropy and diversity. Oikos 113:363–375. DOI: https://doi.org/10.1111/j.2006.0030-1299.14714.x

Kõljalg, U., R.H. Nilsson, K. Abarenkov, … K.H. Larsson. 2013. Towards a unified paradigm for sequence-based identification of fungi. Mol. Ecol. DOI: https://doi.org/10.1111/mec.12481

Lüdecke, D. 2021. sjPlot: Data Visualization for Statistics in Social Science.

McMurdie, P.J., and S. Holmes. 2013. Phyloseq: An R Package for Reproducible Interactive Analysis and Graphics of Microbiome Census Data. PLoS One 8. DOI: https://doi.org/10.1371/journal.pone.0061217

Oksanen, J., F. Blanchet, M. Friendly, … H. Wagner. 2018. Vegan: Community Ecology Package. R package version 2.5-2.

Quast, C., E. Pruesse, P. Yilmaz, J. Gerken, T. Schweer, P. Yarza, J. Peplies, and F.O. Glöckner. 2013. The SILVA ribosomal RNA gene database project: Improved data processing and web-based tools. Nucleic Acids Res. 41:590–596. DOI: https://doi.org/10.1093/nar/gks1219

Segata, N., J. Izard, L. Waldron, D. Gevers, L. Miropolsky, W.S. Garrett, and C. Huttenhower. 2011. Metagenomic biomarker discovery and explanation. Genome Biol. 12.

Toju, H., A.S. Tanabe, S. Yamamoto, and H. Sato. 2012. High-coverage ITS primers for the DNA-based identification of ascomycetes and basidiomycetes in environmental samples. PLoS One DOI: https://doi.org/10.1371/journal.pone.0040863

White, T.J., T. Bruns, S. Lee, and J. Taylor. 1990. Amplification and Direct Sequencing of Fungal Ribosomal Rna Genes for Phylogenetics. PCR Protoc. 315–322. DOI: https://doi.org/10.1016/b978-0-12-372180-8.50042-1

SUPPLEMENTARY FIGURES

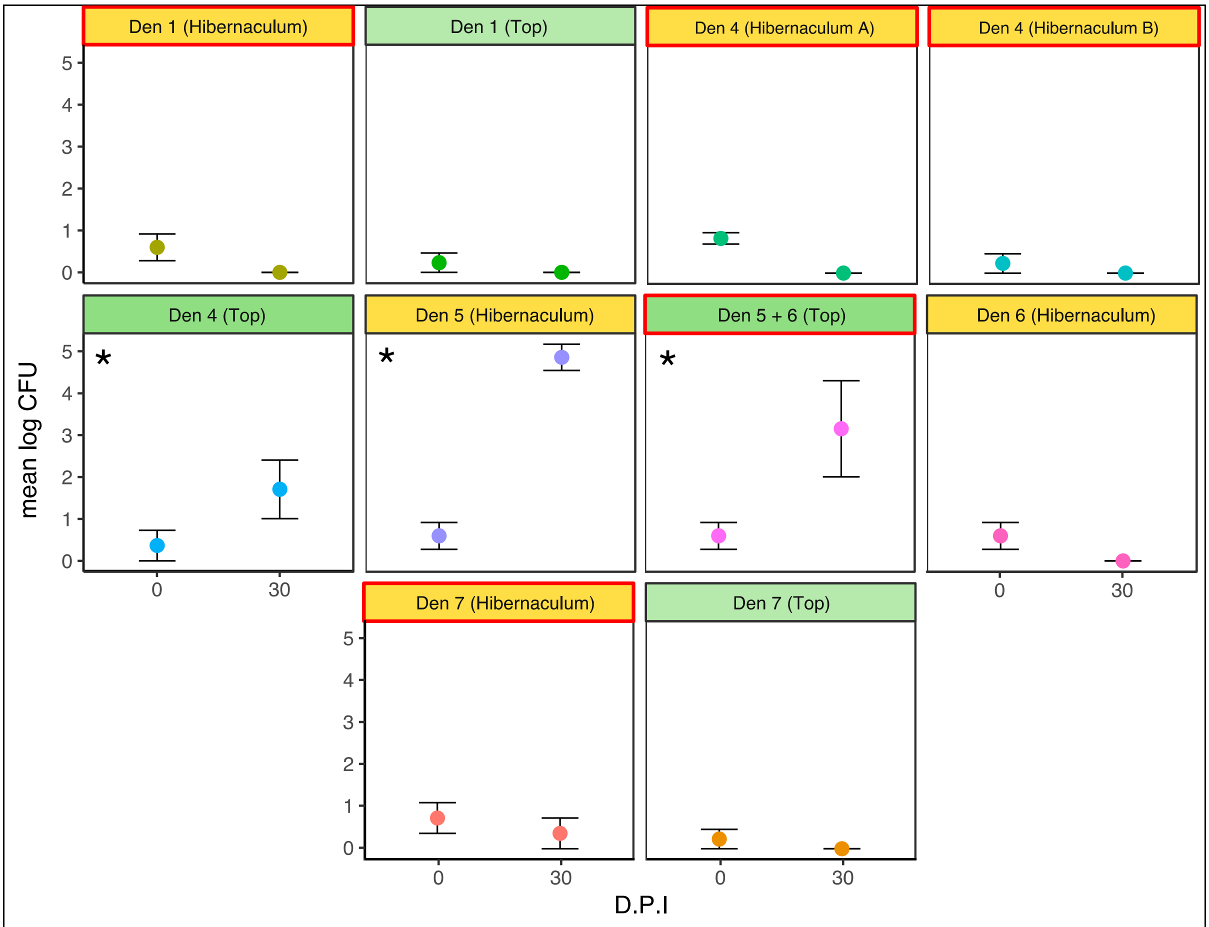

Figure S1. - Colony forming unit (CFU) counts of *Ophidiomyces ophidiicol*a (Oo) in sterile experimental microcosms. Green-headed plots represent topsoil samples; yellow-headed plots represent hibernaculum soils. Red-header borders denote soils that were qPCR positive for Oo. The Y-axis is CFU count on a log scale. The X-axis is days post-inoculation (DPI). Asterix denote soils in which significant increase in Oo CFU counts occurred.

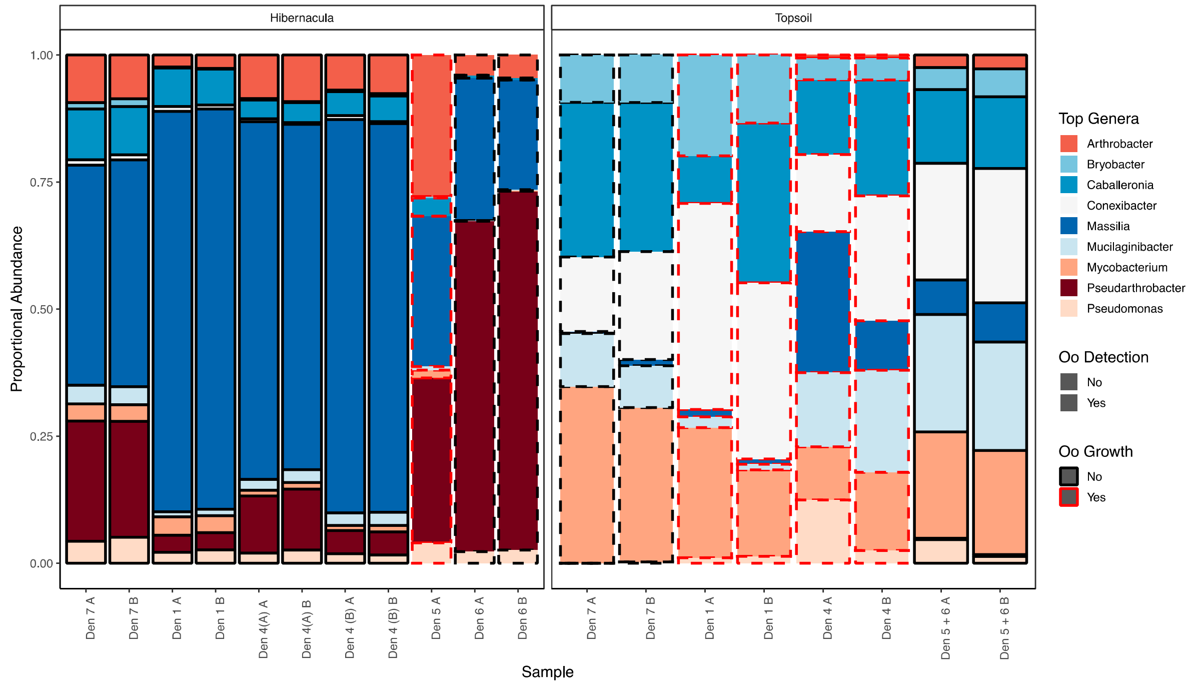

Figure S2.- Proportional abundance of the most abundant bacterial genera per each experimental soil. Each soil is represented twice as each soil was extracted and sequenced in duplicate. Soils with dashed borders demonstrated quantifiable *Ophidomyces ophidiicola* (Oo) growth in our experimental microcosms. Soils with red borders were qPCR positive for Oo. The left panel shows the hibernaculum samples sampled from each den and the right panel shows the associated topsoil samples.

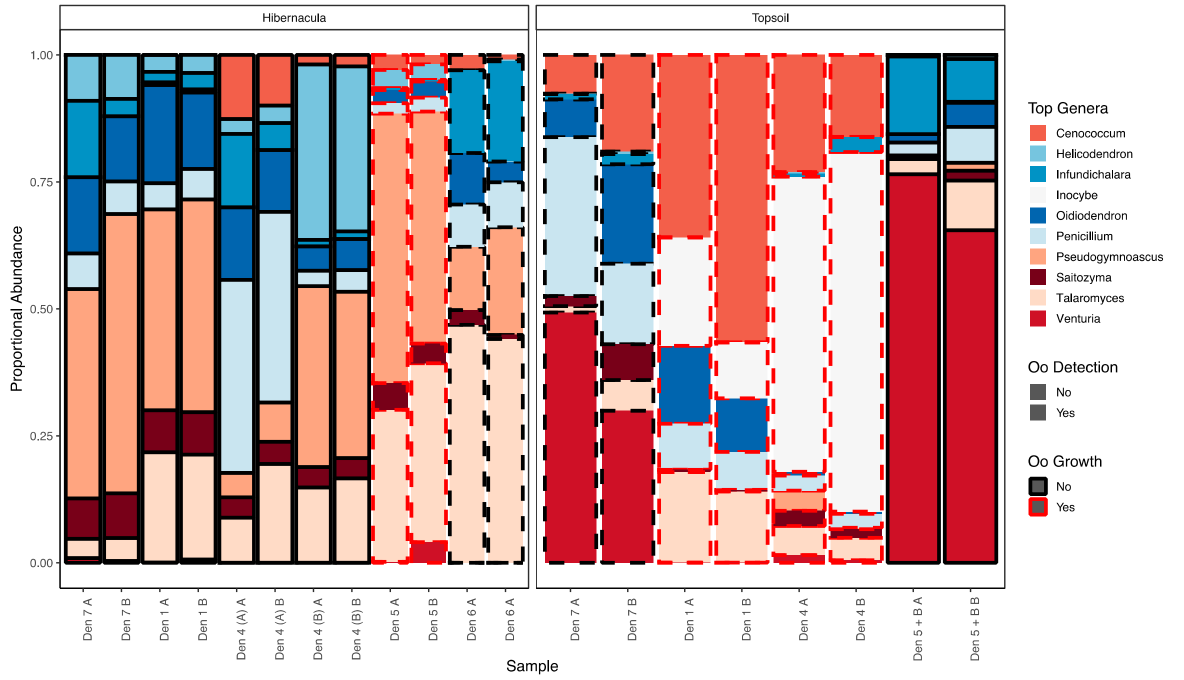

Figure S3.- Proportional abundance of the top 10 most abundant fungal genera per each experimental soil. Each soil is represented twice as each soil was extracted and sequenced in duplicate. Soils with dashed borders demonstrated quantifiable *Ophidomyces ophidiicola* (Oo) growth in our experimental microcosms. Soils with red borders were qPCR positive for Oo. The left panel shows the hibernaculum samples sampled from each den and the right panel shows the associated topsoil samples.

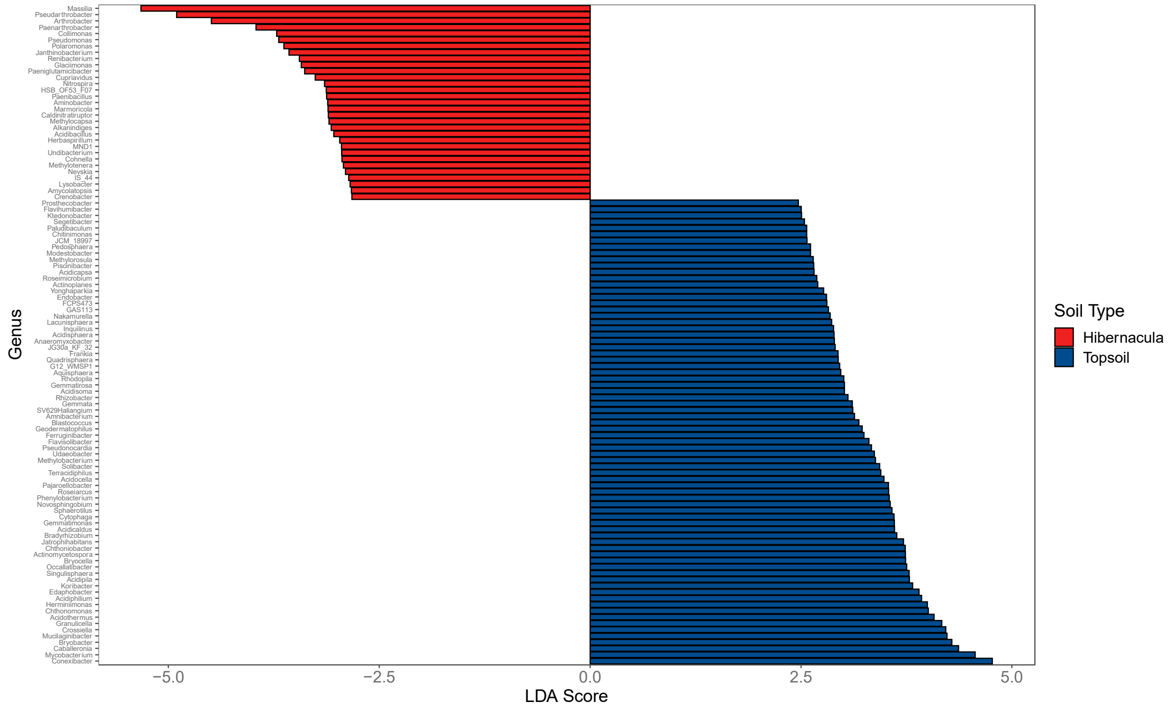

Figure S4.- Linear discriminant effect size (LEfSe) analysis results showing bacterial sequence variants determined to be biomarkers between hibernaculum soils (red) and topsoils (blue). Analysis was performed using the LEfSe glaxay portal with standard parameters.

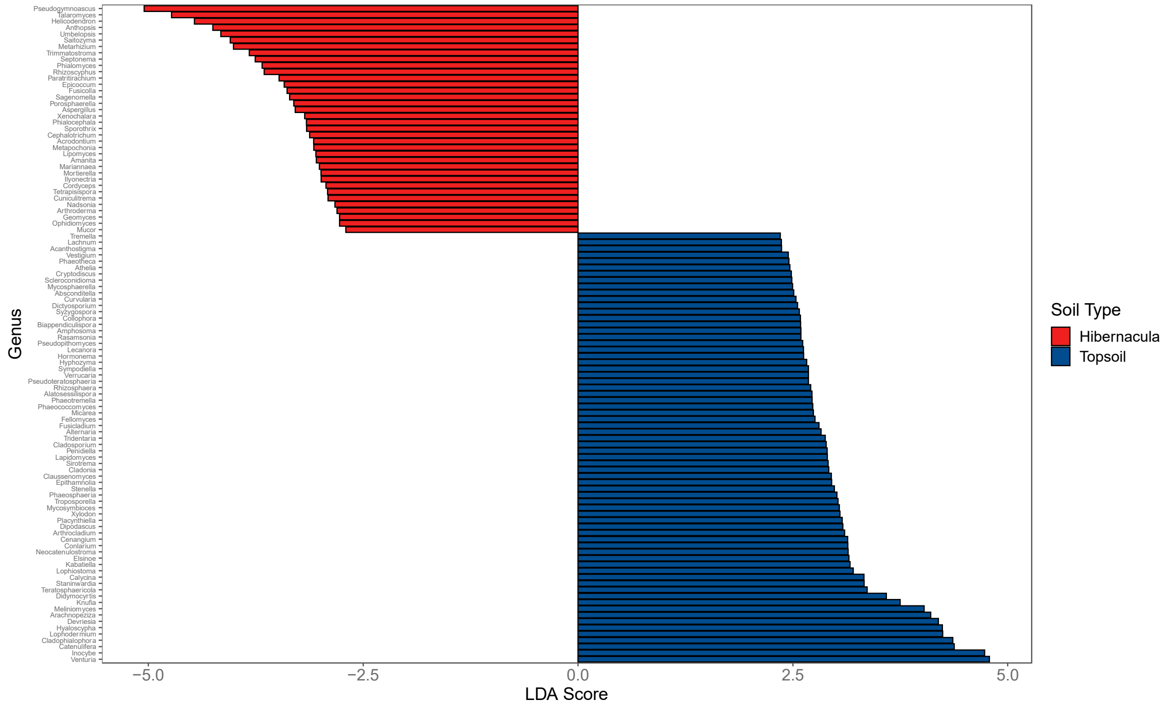

Figure S5.- Linear discriminant effect size (LeFSe) analysis results showing fungal sequence variants determined to be biomarkers between hibernaculum soil (red) and topsoils (blue). Analysis was performed using the LEfSe galaxy portal with standard parameters.

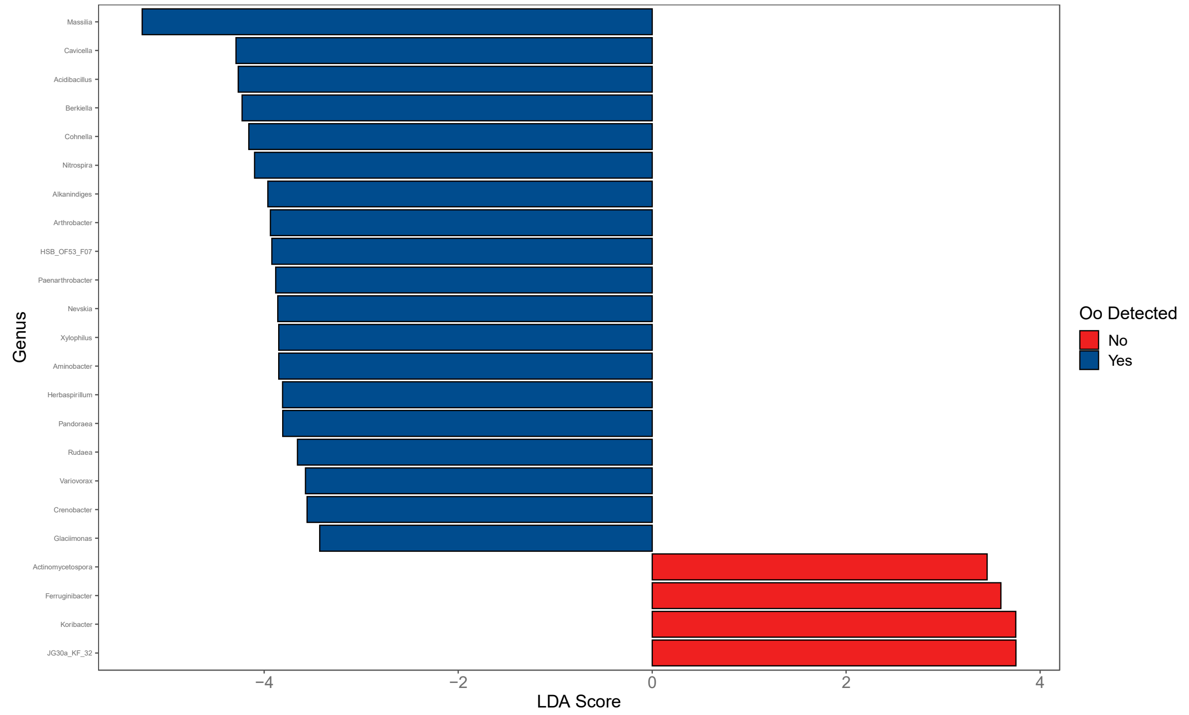

Figure S6.- Linear discriminant effect size (LeFSe) analysis results showing bacterial sequence variants determined to be biomarkers between soils in which *Ophidomyces ophidiicola* (Oo) was detected (blue) and those in which it was not (red). Analysis was performed using the LEfSe galaxy portal with standard parameters.

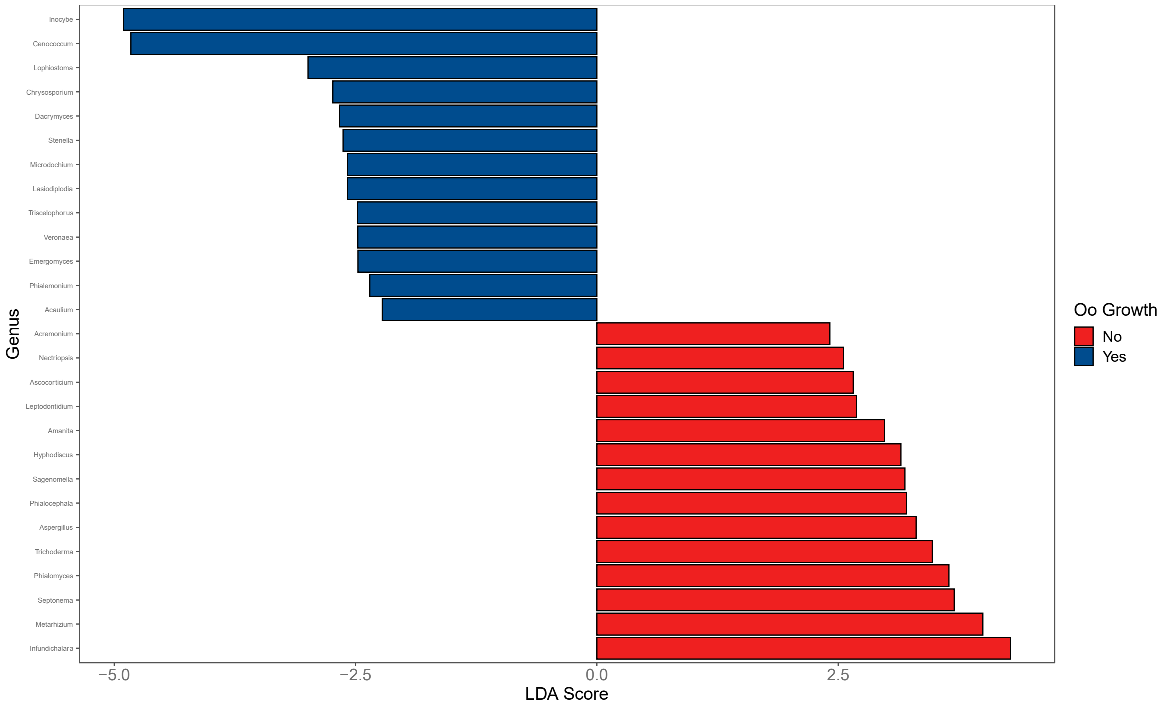

Figure S7: Linear discriminant effect size (LeFSe) analysis results showing fungal sequence variants determined to be biomarkers between soils which inhibited *Ophidomyces ophidiicola* (Oo) growth (red) and those which did not (blue). Analysis was performed using the LEfSe galaxy portal with standard parameters.

SUPPLEMENTARY TABLES

Table S1.- Colony forming unit (CFU) counts of *Ophiomyces ophidiicola* in sterile experimental microcosms. Soils were suspended in 1000ul of phosphate-buffered saline with 0.5% Tween 20 (PBST) and serially diluted from 10^{-1} to 10^{-3}. For each resulting dilution, 100ul was plated onto dermatophyte test medium (DTM) agar plates. Plates were incubated for 15 days at 24°C in the dark at which point Oo CFUs were enumerated.

Den	Type	Day	CFU	Dilution	Replicate
1	Topsoil	0	2	1	A
1	Topsoil	0	0	2	A
1	Topsoil	0	0	3	A
1	Topsoil	0	1	1	B
1	Topsoil	0	0	2	B
1	Topsoil	0	0	3	B
1	Topsoil	0	1	1	C
1	Topsoil	0	0	2	C
1	Topsoil	0	0	3	C
1	Topsoil	30	0	1	A
1	Topsoil	30	0	2	A
1	Topsoil	30	0	3	A
1	Topsoil	30	0	1	B
1	Topsoil	30	0	2	B
1	Topsoil	30	0	3	B
1	Topsoil	30	0	1	C
1	Topsoil	30	0	2	C
1	Topsoil	30	0	3	C
1	Hibernaculum	0	2	1	A
1	Hibernaculum	0	0	2	A

1	Hibernaculum	0	0	3	A
1	Hibernaculum	0	3	1	B
1	Hibernaculum	0	0	2	B
1	Hibernaculum	0	0	3	B
1	Hibernaculum	0	1	1	C
1	Hibernaculum	0	0	2	C
1	Hibernaculum	0	0	3	C
1	Hibernaculum	30	0	1	A
1	Hibernaculum	30	0	2	A
1	Hibernaculum	30	0	3	A
1	Hibernaculum	30	0	1	B
1	Hibernaculum	30	0	2	B
1	Hibernaculum	30	0	3	B
1	Hibernaculum	30	0	1	C
1	Hibernaculum	30	0	2	C
1	Hibernaculum	30	0	3	C
4	Topsoil	0	3	1	A
4	Topsoil	0	0	2	A
4	Topsoil	0	0	3	A
4	Topsoil	0	0	1	B
4	Topsoil	0	0	2	B

4	Topsoil	0	0	3	B
4	Topsoil	0	1	1	C
4	Topsoil	0	0	2	C
4	Topsoil	0	0	3	C
4	Topsoil	30	4	1	A
4	Topsoil	30	0	2	A
4	Topsoil	30	0	3	A
4	Topsoil	30	21	1	B
4	Topsoil	30	6	2	B
4	Topsoil	30	1	3	B
4	Topsoil	30	2	1	C
4	Topsoil	30	0	2	C
4	Topsoil	30	0	3	C
4A	Hibernaculum	0	2	1	A
4A	Hibernaculum	0	1	2	A
4A	Hibernaculum	0	1	3	A
4A	Hibernaculum	0	2	1	B
4A	Hibernaculum	0	0	2	B
4A	Hibernaculum	0	0	3	B
4A	Hibernaculum	0	3	1	C
4A	Hibernaculum	0	0	2	C

4A	Hibernaculum	0	0	3	C
4A	Hibernaculum	30	0	1	A
4A	Hibernaculum	30	0	2	A
4A	Hibernaculum	30	0	3	A
4A	Hibernaculum	30	0	1	B
4A	Hibernaculum	30	0	2	B
4A	Hibernaculum	30	0	3	B
4A	Hibernaculum	30	0	1	C
4A	Hibernaculum	30	0	2	C
4A	Hibernaculum	30	0	3	C
4B	Hibernaculum	0	1	1	A
4B	Hibernaculum	0	0	2	A
4B	Hibernaculum	0	1	3	A
4B	Hibernaculum	0	1	1	B
4B	Hibernaculum	0	0	2	B
4B	Hibernaculum	0	0	3	B
4B	Hibernaculum	0	2	1	C
4B	Hibernaculum	0	0	2	C
4B	Hibernaculum	0	0	3	C
4B	Hibernaculum	30	0	1	A
4B	Hibernaculum	30	0	2	A

4B	Hibernaculum	30	0	3	A
4B	Hibernaculum	30	0	1	B
4B	Hibernaculum	30	0	2	B
4B	Hibernaculum	30	0	3	B
4B	Hibernaculum	30	0	1	C
4B	Hibernaculum	30	0	2	C
4B	Hibernaculum	30	0	3	C
5	Hibernaculum	0	1	1	A
5	Hibernaculum	0	1	2	A
5	Hibernaculum	0	0	3	A
5	Hibernaculum	0	2	1	B
5	Hibernaculum	0	1	2	B
5	Hibernaculum	0	0	3	B
5	Hibernaculum	0	3	1	C
5	Hibernaculum	0	1	2	C
5	Hibernaculum	0	1	3	C
5	Hibernaculum	30	200	1	A
5	Hibernaculum	30	67	2	A
5	Hibernaculum	30	7	3	A
5	Hibernaculum	30	152	1	B
5	Hibernaculum	30	57	2	B

5	Hibernaculum	30	5	3	B
5	Hibernaculum	30	70	1	C
5	Hibernaculum	30	16	2	C
5	Hibernaculum	30	1	3	C
6	Hibernaculum	0	3	1	A
6	Hibernaculum	0	0	2	A
6	Hibernaculum	0	0	3	A
6	Hibernaculum	0	2	1	B
6	Hibernaculum	0	0	2	B
6	Hibernaculum	0	0	3	B
6	Hibernaculum	0	1	1	C
6	Hibernaculum	0	0	2	C
6	Hibernaculum	0	0	3	C
6	Hibernaculum	30	0	1	A
6	Hibernaculum	30	0	2	A
6	Hibernaculum	30	0	3	A
6	Hibernaculum	30	0	1	B
6	Hibernaculum	30	0	2	B
6	Hibernaculum	30	0	3	B
6	Hibernaculum	30	0	1	C
6	Hibernaculum	30	0	2	C

6	Hibernaculum	30	0	3	C
5 + 6	Topsoil	0	3	1	A
5 + 6	Topsoil	0	0	2	A
5 + 6	Topsoil	0	0	3	A
5 + 6	Topsoil	0	1	1	B
5 + 6	Topsoil	0	0	2	B
5 + 6	Topsoil	0	0	3	B
5 + 6	Topsoil	0	2	1	C
5 + 6	Topsoil	0	0	2	C
5 + 6	Topsoil	0	0	3	C
5 + 6	Topsoil	30	200	1	A
5 + 6	Topsoil	30	0	2	A
5 + 6	Topsoil	30	1	3	A
5 + 6	Topsoil	30	16	1	B
5 + 6	Topsoil	30	0	2	B
5 + 6	Topsoil	30	0	3	B
5 + 6	Topsoil	30	4	1	C
5 + 6	Topsoil	30	0	2	C
5 + 6	Topsoil	30	0	3	C
7	Topsoil	0	0	1	A
7	Topsoil	0	0	2	A

7	Topsoil	0	0	3	A
7	Topsoil	0	2	1	B
7	Topsoil	0	1	2	B
7	Topsoil	0	0	3	B
7	Topsoil	0	0	1	C
7	Topsoil	0	0	2	C
7	Topsoil	0	0	3	C
7	Topsoil	30	0	1	A
7	Topsoil	30	0	2	A
7	Topsoil	30	0	3	A
7	Topsoil	30	0	1	B
7	Topsoil	30	0	2	B
7	Topsoil	30	0	3	B
7	Topsoil	30	0	1	C
7	Topsoil	30	0	2	C
7	Topsoil	30	0	3	C
7	Hibernaculum	0	3	1	A
7	Hibernaculum	0	0	2	A
7	Hibernaculum	0	0	3	A
7	Hibernaculum	0	3	1	B
7	Hibernaculum	0	0	2	B

7	Hibernaculum	0	0	3	B
7	Hibernaculum	0	1	1	C
7	Hibernaculum	0	0	2	C
7	Hibernaculum	0	0	3	C
7	Hibernaculum	30	0	1	A
7	Hibernaculum	30	0	2	A
7	Hibernaculum	30	0	3	A
7	Hibernaculum	30	3	1	B
7	Hibernaculum	30	0	2	B
7	Hibernaculum	30	0	3	B
7	Hibernaculum	30	0	1	C
7	Hibernaculum	30	0	2	C
7	Hibernaculum	30	0	3	C

Table S2.- Results of soil abiotic parameter analysis and associated metadata. Each soil sample was split into two, and one group of each was sterilised via autoclaving. Samples were sent to the University of Wisconsin's Soil Sciences extension for analysis. Results columns prefixed with "Sterile" present the results for soil samples which were sterilised before analysis. "NonSterile" refers to soils which were not sterilised prior to analysis.

Den	Type	qPCR_Growth	CFU_Growth	Oo_Detected	NonSterile_pH	Sterile_pH
Den 1	Topsoil	Yes	No	No	5.8	4.1
Den 1	Hibernaculum	No	No	Yes	5.2	4.2
Den 4	Topsoil	Yes	Yes	No	5.8	4.3
Den 4 A	Hibernaculum	No	No	Yes	4.9	3.9
Den 4 B	Hibernaculum	No	No	Yes	4.3	3.9
Den 5 + 6	Topsoil	Yes	Yes	Yes	4.3	3.9
Den 5	Hibernaculum	Yes	Yes	No	6	5
Den 6	Hibernaculum	No	No	No	5.4	4.3

Den 7	Topsoil	No	No	No	4.9	3.7
Den 7	Hibernaculum	No	No	Yes	4.9	3.9

Table S2 Cont.

Den	NonSterile_P	Sterile_P	NonSterile_K	Sterile_K	NonSterile_OM	Sterile_OM
Den 1	5	6	3	5	0.7	0.8
Den 1	5	5	3	3	0.4	0.5
Den 4	14	17	5	8	1.1	1.4
Den 4 A	15	15	1	1	0.3	0.3
Den 4 B	21	18	1	1	0.4	0.3
Den 5 + 6	6	11	2	5	0.7	1
Den 5	6	5	2	2	0.4	0.3
Den 6	6	12	1	1	0.2	0.2
Den 7	5	10	6	11	2.4	2.2
Den 7	7	7	1	2	0.6	0.7

Table S3. - PCR cycling conditions used in the amplification of next generation sequencing amplicons

16S Amplification

Step	Temp	Time	Cycles
1	95°C	15 minutes	1
2	94°C	30 seconds	35
3	58°C	30 seconds	35
4	72°C	1 minute	35
5	72°C	10 minutes	1

Fungal ITS2 Amplification

Step	Temp	Time	Cycles
1	95°C	15 minutes	1
2	94°C	30 seconds	35
3	56°C	30 seconds	35
4	72°C	1 minute	35
5	72°C	10 minutes	1
